# Supplementary material for: Moiré metrology of energy landscapes in van der Waals heterostructures
Source: Nat Commun. 2021 Jan 11;12:242. doi: 10.1038/s41467-020-20428-1 (PMC7801382; doi:10.1038/s41467-020-20428-1)
Supplement: Supplementary file 2 — Description of Additional Supplementary Files [file 41467_2020_20428_MOESM2_ESM.pdf]

Description of Additional Supplementary Files

Title: Supplementary Movie 1

Description: Single tuning parameter modelling of twisted bilayer graphene domain walls. The video presents the fits for different choices of the single tuning parameter β to the TBG domain structures (see SI section S4), covering the range between $\hat{\beta}$= 2.00, where the AA pinning sites are connected by straight SDWs, to $\hat{\beta}$= 1.70, where all SDWs collapse to DDWs intersecting at the Fermat point of the triangle. The positions of the AA pinning sites were manually set to match the experimental data of Fig. 2a.

Title: Supplementary Movie 2:

Description: Domain formation in the microscopic model and 2D soap-bubble model of TDBG – the DFT-D2 approach. The video presents a series of simulations of the rhombohedral domain formation in TDBG as a function of twist angle, covering all presented domain structure regimes. The false color corresponds to the stacking energy density. The solution was obtained using the full 2D relaxation model presented at SI section S1 above, with symmetry conserving boundary conditions along the high symmetry lines of the GSFE. The superimposed green dashed lines correspond to the domain shape from the 2D soap-bubble model (section S7), with fixed σ, ET,SDW, ES,SDW, γ2 parameters, which were extracted directly from other calculations: σ directly extracted from the GSFE, ET,SDW,ES,SDW from the resulting elliptic shape of full 2D relaxation simulation as presented in Fig. 3c-d, and γ2 from 1D domain wall structure calculations as described in SI section S1 above. This video used the GSFE of the DFT-D2 approach, as listed in Table 1 (SI section S2).

Title: Supplementary Movie 3

Description: Domain formation in the microscopic model and 2D soap-bubble model of TDBG – the LDA approach. Same as for Supplementary Video 2, except for using the GSFE of the LDA approach, as listed in Table 1.

Title: Supplementary Movie 4

Description: Domain formation in the microscopic model and 2D soap-bubble model of TDBG – the GGA-TS approach. Same as for Supplementary Video 2, except for using the GSFE of the GGA-TS approach, as listed in Table 1.

Title: Supplementary Movie 5

Description: Domain formation in the microscopic model and 2D soap-bubble model of TDBG – the optB88-vdW approach. Same as for Supplementary Video 2, except for using the GSFE of the optB88-vdW approach, as listed in Table 1.

Title: Supplementary Movie 6

Description: Strain effect on TBG moiré super-lattice. SI section S8 analyzes the effect of strain on the unit cell of a moiré super-lattice. For completeness, this video shows the stacking energy density from 2D relaxation calculation for twisted bilayer graphene, using the GSFE available in literature31. The video shows how in the extreme strain case DDWs form as well, as the moiré super-lattice approaches a 1D structure. In these calculation a Poisson ratio of 0.22 was used, and the lattice is strained along the horizontal
