# Supplementary material for: Moiré metrology of energy landscapes in van der Waals heterostructures
Source: Nat Commun. 2021 Jan 11;12:242. doi: 10.1038/s41467-020-20428-1 (PMC7801382; doi:10.1038/s41467-020-20428-1)
Supplement: Supplementary file 3 — Supplementary Information [file 41467_2020_20428_MOESM3_ESM.pdf]

Supplementary Information - Moiré metrology of energy landscapes in van der Waals heterostructures

Dorri Halbertal[1*], Nathan R. Finney[2], Sai S. Sunku[1], Alexander Kerelsky[1], Carmen Rubio-Verdú[1], Sara Shabani[1], Lede Xian[3,4], Stephen Carr[5,6], Shaowen Chen[1,7], Charles Zhang[1,8], Lei Wang[1,9], Derick Gonzalez-Acevedo[1,7], Alexander S. McLeod[1], Daniel Rhodes[1,10], Kenji Watanabe[11], Takashi Taniguchi[11], Efthimios Kaxiras[12], Cory R. Dean[1], James C. Hone[2], Abhay N. Pasupathy[1], Dante M. Kennes[3,13], Angel Rubio[3,14], D. N. Basov[1]

S 1. Relaxation models

In this work two real space simulation tools have been developed for the solution of the relaxation problem, a 1D and a full 2D code. In both cases the energy functional depends on position, the displacement field of each layer and their spatial gradients, and is composed of an elastic energy term and a stacking energy term. These models assume that the inter-layer displacement field changes on a length-scale larger than α, the atomic unit-cell spacing. As a result, the local stacking energy density is assumed to be a direct consequence of the generalized stacking fault energy function (GSFE), by assuming that the local stacking energy density is the same as for an infinite bulk arrangement of the same stacking configuration. The total energy would be $E = \int d^2r\mathcal{E}(\boldsymbol{r},\boldsymbol{u},\boldsymbol{\nabla u})$ where the energy functional in its 2D spatial coordinate form is:

$$\mathcal{E}(\boldsymbol{r},\boldsymbol{u},\boldsymbol{\nabla u}) = \mathcal{E}_{elastic}(\boldsymbol{\nabla u}) + \mathcal{E}_{stacking}(\boldsymbol{r},\boldsymbol{u})$$

Where $\boldsymbol{u}$ is the inter-layer displacement, and we assume that each layer is displaced such that: $\boldsymbol{u_t} = \mu\boldsymbol{u}, \boldsymbol{u_b} = -(1-\mu)\boldsymbol{u}$ for a dimensionless parameter μ allowing to tune the layer motion of bottom (b) and top (t) layers, where $0 \leq \mu \leq 1$. $\mu = \frac{1}{2}$, for instance, is the case where the two layers are displaced in an anti-symmetric fashion. In the simulations presented in this work we used $\mu = \frac{1}{2}$. The conclusions of

[1]Department of Physics, Columbia University, New York, NY, USA.
[2]Department of Mechanical Engineering, Columbia University, New York, NY, USA.
[3]Max Planck Institute for the Structure and Dynamics of Matter and Center Free-Electron Laser Science, Luruper Chaussee 149, 22761 Hamburg, Germany.
[4]Present address: Songshan Lake Materials Laboratory, Dongguan, Guangdong 523808, China.
[5]Department of Physics, Harvard University, Cambridge, Massachusetts 02138, USA.
[6]Present address: Brown University, Providence, RI 02912, USA.
[7]Present address: Department of Physics, Harvard University, Cambridge, MA 02138, USA.
[8]Present address: Department of Physics, University of California at Santa Barbara, Santa Barbara, CA 93106, USA.
[9]Present address: National Laboratory of Solid-State Microstructures, School of Physics and Collaborative Innovation Center of Advanced Microstructures, Nanjing University, Nanjing, China
[10]Present address: Department of Materials Science and Engineering, University of Winsconsin-Madison, WI 53706, USA.
[11]National Institute for Material Science, Tsukuba, Japan
[12]John A. Paulson School of Engineering and Applied Sciences, Harvard University, Cambridge, Massachusetts 02138, USA.
[13]Institut fur Theorie der Statistischen Physik, RWTH Aachen University, 52056 Aachen, Germany.
[14]Center for Computational Quantum Physics, Flatiron Institute, New York, NY 10010 USA.
*Correspondence and requests for materials should be addressed to D.H. (dh2917@columbia.edu).

the TBG section of the paper do not depend on this choice. For TDBG this choice is justified in Supplementary Information S2 below.

The elastic term has the explicit form:

$$\mathcal{E}_{elastic}(\nabla\boldsymbol{u}) = \frac{1}{2}K\left(\partial_x u_x + \partial_y u_y\right)^2 + \frac{1}{2}G\left(\left(\partial_x u_x - \partial_y u_y\right)^2 + \left(\partial_x u_y + \partial_y u_x\right)^2\right)$$

$$K = (1-\mu)^2 K_b + \mu^2 K_t, G = (1-\mu)^2 G_b + \mu^2 G_t$$

Where K_t (K_b) and G_t (G_b) are the bulk and shear elastic moduli of the top (bottom) layer.

The stacking energy term has the form:

$$\mathcal{E}_{stacking}(\boldsymbol{r},\boldsymbol{u}) = V_{GSFE}\left(v(\boldsymbol{r},\boldsymbol{u}), w(\boldsymbol{r},\boldsymbol{u})\right)$$

Where the GSFE in its general form follows the notation of Ref. 31:

$$\begin{aligned} V_{GSFE}(v,w) = c_0 &+ c_1(\cos v + \cos w + \cos(v+w)) + c_2(\cos(v+2w) + \cos(v-w) + \cos(2v+w)) \\ &+ c_3(\cos 2v + \cos 2w + \cos(2v+2w)) + c_4(\sin v + \sin w - \sin(v+w)) \\ &+ c_5(\sin(2v+2w) - \sin 2w - \sin 2v) \end{aligned}$$

And $\Omega \equiv \begin{pmatrix} v(\boldsymbol{r},\boldsymbol{u}) \\ w(\boldsymbol{r},\boldsymbol{u}) \end{pmatrix}$ tracks the stacking configuration of the two layers. We assume the two layers have a hexagonal lattice with Bravais spacing of $\alpha_b = \alpha, \alpha_t = (1+\delta)\alpha$, where δ is the lattice mismatch. We assume a global twist θ and displacement $\Delta\boldsymbol{r}$ between the layers, such that in absence of additional local displacements the top layer's AA sites will be located at $\frac{v}{2\pi}\boldsymbol{b}_1 + \frac{w}{2\pi}\boldsymbol{b}_2$ for v, w which are integer multiples of 2π where $\boldsymbol{b}_1 = \begin{pmatrix} \cos\theta_0 \\ \sin\theta_0 \end{pmatrix}, \boldsymbol{b}_2 = \begin{pmatrix} \cos(\theta_0 + \frac{\pi}{3}) \\ \sin(\theta_0 + \frac{\pi}{3}) \end{pmatrix}$ are the normalized Bravais vectors of the bottom layer and θ_0 is the orientation of the zigzag direction. For such definitions one can show that the local configuration would be:

$$\Omega(\boldsymbol{r},\boldsymbol{u}) = \frac{2\pi}{\alpha}[\boldsymbol{b}_1 \quad \boldsymbol{b}_2]^{-1}\left((R_{-\theta} - (1+\delta)I_2)\boldsymbol{r} - \left(\mu R_{-\theta} + (1-\mu)(1+\delta)\right)\boldsymbol{u} - R_{-\theta}\Delta\boldsymbol{r}\right)$$

Where $R_{-\theta} = \begin{pmatrix} \cos\theta & \sin\theta \\ -\sin\theta & \cos\theta \end{pmatrix}$ is a rotation matrix by the global twist angle and I_2 is the 2D identity matrix. For the case of a homo-bilayer system and assuming a fixed bottom layer without global translation, the expression can be simplified to be:

$$\Omega(\boldsymbol{r},\boldsymbol{u}) = \frac{2\pi}{\alpha}[\boldsymbol{b}_1 \quad \boldsymbol{b}_2]^{-1}\left((R_{-\theta} - I_2)\boldsymbol{r} - R_{-\theta}\boldsymbol{u}\right)$$

The remaining component for a well-defined problem is the boundary conditions (BC). For the 1D case, used for solving for the structure of single (SDW) and double (DDW) domain walls in the discussed systems, we assume u_x and u_y depend only on x (which further simplifies the expressions), and assume a predefined boundary conditions for the stacking configurations on both sides of the domain wall dislocation. In such an approach we can describe both SDW (as an AB/BA interface) and DDW (as an interface between AB and AB across a BA segment in configuration space). From such calculations we can extract the energy costs per unit length angular functions $\gamma_1(\varphi)$ (for TBG) and $\gamma_2(\varphi)$ (for TBG and TDBG).

These functions have a π periodicity, and can be approximated as $\gamma_j(\varphi) = \left(E_{S,j}^n \cos^2\varphi + E_{T,j}^n \sin^2\varphi\right)^{\frac{1}{n}}, j = SDW, DDW$ where φ is the angle relative to the shear direction of the dislocation. Note that in the limit $n \to 0$, the expression converges to $\gamma_j(\varphi) = E_{S,j}\left(\frac{E_{T,j}}{E_{S,j}}\right)^{\sin^2\varphi}$. The fitted parameters, following such 1D calculations, which were used in the analysis throughout this work are detailed in table 1. The SDW in the case of TDBG is not a stable 1D soliton structure, therefore one needs a full 2D solution in order to extract $\gamma_1(\varphi)$ for TDBG as done in Supplementary Information S7 and summarized in Table 1 (Supplementary Information S2).

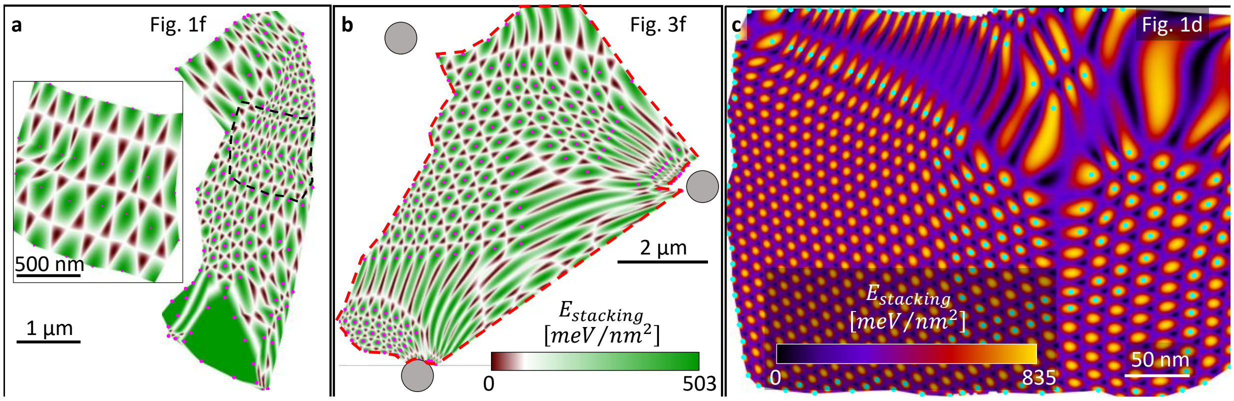

Supplementary Figure S1|Initial and boundary conditions for simulations in this work. Colored dots mark points used to impose a given stacking configuration. False color map corresponds to stacking energy density resulting from the interpolation of the displacement field between these points. **a,** The TDBG case presented in Fig. 1c. Inset corresponds to modified initial conditions that warps the moiré dislocation from the other side (in region marked by dashed frame). Fig. 1f is composed of a combination of the two relaxation calculations. **b,** The TDBG case presented in Fig. 3f. **c,** The $MoSe_2/WSe_2$ case presented in Fig. 1g. **a** and **b** share a color-map.

The boundary conditions in the 2D cases provided throughout the paper are more involved. They include rigid pre-defined stacking at selected points (as in Fig. 1f-g, Fig. 2d-e and Fig. 3f of the main text) and periodic boundary conditions (as in Fig. 1a,e and Figs. 3c-d of the main text). In the rigid BC case we impose the stacking configuration at selected points, while the periodic boundary conditions impose the periodicity of the moiré super-lattice in the displacement field, possibly with an additional uniform external strain.

The energy functional was minimized with standard unconstrained minimization techniques (trust region algorithm), using an interpolation between the BC points as an initial guess (as detailed in next section). The simulation tools used in this work were verified against calculations in Ref. 31, and with other results generated by the same code, which was courteously provided by the authors of Ref. 31.

S 2. Details of relaxation calculations and material properties

For completeness, we provide details of 2D relaxation calculations performed throughout this work. Fig. 1a: The atomic positions in the sketch of Fig. 1a were calculated using a periodic relaxation calculation. The TDBG calculation (top) used the DFT-D2 model for a twist angle of 3° (moiré period of 4.72 nm), but with an interlayer coupling increased by a factor of $2 \cdot 10^4$, as to induce stronger curvature

and formation of DDW. The T-H-$MoSe_2/WSe_2$ (bottom) used the GSFE detailed in Table 1 (Supplementary Information S2), with a twist angle of 3.989° (to have similar moiré period as in the TDBG case) and an interlayer coupling increased by a factor of 25 (similarly motivated as in the TDBG case). Fig. 1e: For Fig. 1e a twist angle of 0.081° and an additional small external strain of 0.026% using a Poisson ratio of $\nu = 0.22$ was introduced, in order to model the experimental results of Fig. 1b. Fig. 1f, 1d, 3f, and Supplementary Fig. S9b: The simulation boundary and initial conditions (the initial guess used in the optimization process) for these calculations are shown in Supplementary Figure S1. The used GSFE parameters for each material are listed in Table 1 (DFT-D2 approach for TDBG). For each simulation a

Material	TBG[31,34]	TBG - mod. $\tau = 0.025$ $\zeta = 0.3$	TDBG (DFT-D2)	TDBG (LDA)	TDBG (GGA-TS)	TDBG (optB88-vdW)	$MoSe_2/WSe_2$ 180° twist	
K	69518	69518	139036	139036	139036	139036	40521	43113
G	47352	47352	94704	94704	94704	94704	26464	30770
c_0	6.832	6.1891	10.4395	7.6484	9.7361	7.7155	42.6	
c_1	4.064	3.2041	6.0761	4.3773	5.861	4.6825	16.0	
c_2	-0.374	-0.07855	-0.4995	-0.4088	-0.3330	-0.2712	-2.7	
c_3	-0.095	0.76486	-0.1972	-0.1384	-0.0771	-0.0989	-1.1	
c_4	0	0	0.0453	0.02196	0.0141	0.0041	3.7	
c_5	0	0	0.0019	0.0025	0.0093	0.0060	0.6	
$E_{S,SDW}$	953	1201	1103	965	1062	928	N/A	
$E_{T,SDW}$	1497	1887	1776	1531	1681	1460		
n_{SDW}*	1.49	1.04	2**	2**	2**	2**		
$E_{S,DDW}$	2184	2606	2609	2267	2492	2171		
$E_{T,DDW}$	2744	3433	3339	2890	3170	2747		
n_{DDW}*	0.72	4.16	1.43	1.34	1.266	1.01		
σ	0	0	4.637	2.404	2.309	0.997		

Table 1: Material coefficients (above bold line) and extracted parameters (below bold line) used in this work. All material coefficients have units of $meV/u.c.$, all graphene heterostructures use a unit-cell (u.c.) spacing of $\alpha = 0.247\ nm$, while $\alpha = 0.3288\ nm$ for $MoSe_2$ and $\alpha = 0.3282\ nm$ for WSe_2. K, G are the bulk and shear moduli (for $MoSe_2/WSe_2$ hetero-bilayer the values for each layer are mentioned separately), and $c_0 - c_5$ are the generalized stacking fault energy function (GSFE) coefficients, following the notation of Ref. 31. For TBG: $K, G, c_0 - c_5$ are taken from Ref. 31. "TBG – mod. $\tau = 0.025, \zeta = 0.3$" denotes the moiré constrained version of the parameters of TBG of Ref. 31 in order to reproduce experimental formations of DDWs (see Fig. 2 and discussion in Supplementary Information S4). The GSFE parameters of TDBG were calculated using different DFT approaches (methods for more details), while K, G were simply taken as twice that of TBG. $E_{S,SDW}$, $E_{T,SDW}$, $E_{S,DDW}$ and $E_{T,DDW}$ are in units of meV/nm and σ in meV/nm^2. *Exponents are fitted to provide analytic expression approximating the calculated angular curves. **SDW parameters for TDBG were extracted from fitting an elliptic arc to the SDW as discussed in Supplementary Information S7, therefore assuming $n_{SDW} = 2$.

given configuration was imposed at the points marked by circles. The false color maps represent the initial condition that was set by spline interpolation of the displacement field in between these points. Fig. 1f required a special treatment due to the existence of a dislocation in the moiré superlattice (see dashed circle in Fig. 1f). In order to account for the dislocation in the stacking energy density map, two calculations were performed one covered by the panel of Supplementary Fig. S1a, and the other by the inset of Supplementary Fig. S1a that covers the dislocation from the other side. Stitching the two images allow to have some account of the dislocation, even though the presented model does not implicitly support such cases. Fig. 2d-e are covered in Supplementary Information S4 below. Fig. 3c-d Fig. S6b and Supplementary Videos 2-5 assumed zero external strain, and were solved with periodic boundary conditions. Figs. S2d-f and Supplementary Video 6 were solved with periodic boundary conditions and a uniform external strain tensor as indicated in Supplementary Information S8. The calculation of Supplementary Fig. S6a is discussed in detail in Supplementary Information S7.

In all calculation we used $\mu = 0.5$ (see Supplementary Information S1 above), that distributes the displacement equally between the two layers of the calculation. While this choice makes physical sense, and has been commonly used in literature as well[31], it is in fact supported by the experimental results presented in Fig. 3 for TDBG. Deviating from $\mu = 0.5$ makes the layers effectively stiffer, thus increasing the line energies of a domain wall. Since the ABCA stacking energy density dependence only on the GSFE, changing μ away from this value will lead to an increase in κ^{-1}. Therefore, the comparison of the histogram of Fig. 3a with relaxation calculations of the 4 explored DFT approaches supports the choice $\mu = 0.5$.

The material properties that were used in the relaxation calculations are summarized in Table 1 (of different DFT approaches from literature where referenced, and otherwise calculated within this work). Relevant extracted parameters are also mentioned (below the bold lines).

S 3. Evaluation of the generalized stacking fault energy function of MoSe2/WSe2

The map of Fig. 1d reveals the formation of double domain walls (DDWs) in strained region of the twisted H-stacked $MoSe_2/WSe_2$ structure. Here we compare the experimental DDW structure with the relaxation solution using calculated generalized stacking fault energy function (GSFE – see Table 1 in Supplementary Information S2). Supplementary Fig. S3 presents another STM scan (constant current mode) of a similar sample, showing a series of parallel formation of DDWs. The line-cut (inset) reveals a full-width-half-maximum DDW width of 3.5 nm. A 1D relaxation calculation was performed using the calculated parameters for $MoSe_2/WSe_2$ of Table 1 of Supplementary Information S2 to resolve the calculated DDW structure. In order for the DDW to be a well-defined 1D structure, the mismatch had to be artificially removed by equating the lattice constant of the two materials (in the 2D calculation, no such simplification was made). The calculated DDW width using the parameters of Table 1 of Supplementary Information S2 is 3.4 nm, with excellent agreement with the experimental observation.

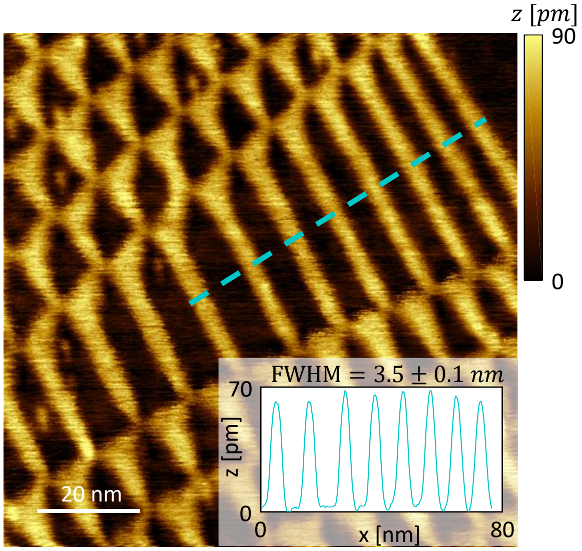

Supplementary Figure S2|H-stacked MoSe2/WSe2 DDW width from STM measurements (constant current mode). Inset: a cross-section of along the dashed marked line, showing a full-width-half-maximum (FWHM) DDW width of 3.5 ± 0.1 nm.

S 4. Study of TBG generalized stacking fault energy function using single vs. double domain wall formation

In this section we provide a detailed account of the twisted bilayer graphene (TBG) analysis presented in the main text. The analysis in this section is based on the non-local photocurrent image of Supplementary Fig. S3a. The non-local photocurrent technique is a novel imaging technique that differ from the more conventional thermo-electric based nano-photocurrent imaging by the origin of the photocurrent signal. In the conventional thermo-electric case, photocurrent is generated through the Seebeck effect as a convolution between the hot spot generated by the enhanced electric field under the tip, and local variations in the Seebeck coefficient. In our case due to the low temperature and high doping the generated temperature profile is broadened (Supplementary Fig. S3b – top left), and the local photocurrent contribution is suppressed. However, due to the existence in our case of an interface between monolayer (MLG) and TBG, which provides a sharp step in Seebeck coefficient (Supplementary Fig. S3b – top right), photocurrent is actually generated at the interface, away and independent of tip position. As a result, the technique is sensitive to local changes in optical conductivity governing local absorption (illustrated by Supplementary Fig. S3b – bottom), and reveals in great details all subcomponents of the structure: from AB and BA domains (orange and cyan dots in Fig. 2a), SDWs and DDWs, AA sites (red dot in Supplementary Fig. S3a) and plasmonic fringes in the MLG.

The single tuning parameter model presented in the main text attempts to capture the essence of the process governing the formation of the network of domain walls as a competition between SDWs and DDWs. It is an oversimplification in the sense that it neglects the angular dependence of the energy cost per unit length of the domain walls, but it is still useful for the discussion. This model reduces the competition between SDWs and DDWs to one ratio, $\bar{\beta}$, of the energy per unit length of a DDW oriented along the shear direction of the DDW dislocation, and the energy per unit length of a SDW oriented along the same direction (of minimal energy for forming DDW). The rationale is that in order to form a DDW,

two SDWs have to be brought together, and bend to reorient along the same direction. This quantity captures the effective attraction between two proximate SDWs, and therefore plays an important role.

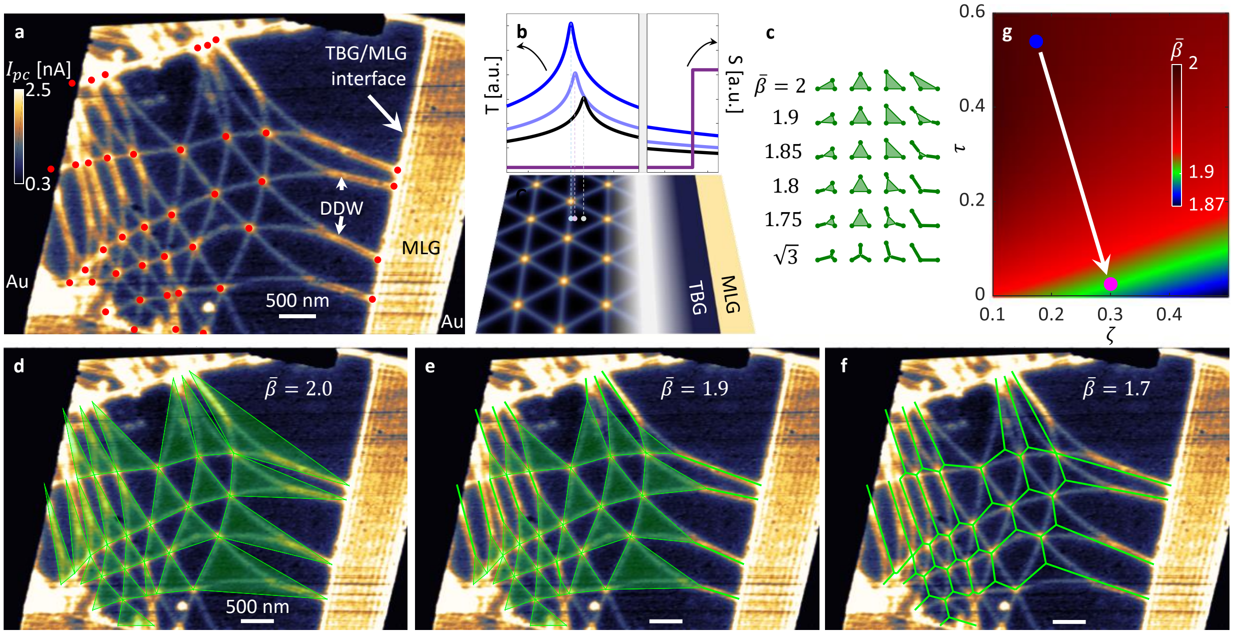

Supplementary Figure S3|Single tuning parameter model for domain formation in TBG. a, Non-local photocurrent map of moiré super-lattice of a TBG sample. Gold contact (marked by Au) on the left is grounded and the one on the right collects current through a trans-impedance current preamplifier. The map is the collected current for a given tip position (see methods for more information). Temperature is 100 K and sample carrier concentration is tuned to $3 \cdot 10^{12}\ \mathrm{cm}^{-2}$. Data reveal the formation of double domain wall (an example is marked by "DDW") separating domains of similar stacking configurations. **b,** Schematic description of the non-local photocurrent imaging technique. The measurement regime leads to a large thermal cooling length (relative to system size) resulting in delocalized temperature profiles (demonstrated by top- panel representing temperature profiles for different tip positions). In addition, the MLG/TBG interface acts as a Seebeck coefficient step (right top panel) and dominates photo-current generation. Non-local photocurrent should be regarded as a measure of local optical conductivity. **c,** Single fitting parameter model ($\bar{\beta}$ – see text for details) describing domain formation as a competition between single (SDW) and double (DDW) domain walls. In each domain the AA sites (triangle vertices) are fixed and SDW (thin lines) can collapse to form DDW (thick lines) to minimize total energy. This panel shows how different triangles (columns) would collapse for selected $\bar{\beta}$ values (rows). **d-f,** Domain formation within the model parameter model for different fitting parameter values (as mentioned) and AA sites position (marked by red dots in **a**), overlaid on top of experimental data. **g,** Mapping of candidates for GSFE of TBG according to the extracted value of $\bar{\beta}$ (calculated by 1D relaxation calculations – see Supplementary Information S1 for more details). Curves of GSFE candidates in Fig. 2b are marked bv similar colors here.

Next we anchor the vertices of a given triangle at the AA sites (red dot in Supplementary Fig. S3a) and minimize the total energy of a triangle by allowing two SDW to collapse and form a segment of a DDW. Supplementary Fig. S3c schematically shows how different triangular arrangements of the AA sites would minimize the energy as a function $\bar{\beta}$. In the trivial case at $\bar{\beta}$=2 (Supplementary Fig. S3c top raw, and similarly the fit of Supplementary Fig. S3d), there is no benefit in forming DDWs, and the minimal energy

would yield triangular domains. In the low $\bar{\beta}$ limit, in particular $\bar{\beta} = \sqrt{3}$ (bottom raw in Supplementary Fig. S3c), all SDW for every triangular geometry would collapse to DDW intersecting at the Fermat point of the triangle (Supplementary Fig. S3f). We can use $\bar{\beta}$ as a fitting parameter and search for the value that would reproduce the experimental network of Fig. 2a (the full span of values is presented in Supplementary Video 1). The predicted network is strongly dependent on the fitting parameter, and the optimal fit is achieved for $\bar{\beta} = 1.90$ (Supplementary Fig. S3e) with surprisingly good agreement with experimental data, considering the oversimplification of these assumptions.

To quantify moiré networks constraints on the stacking energy landscape, we spanned according to the value of $\bar{\beta}$ all possible GSFE's for TBG (satisfying the symmetry of TBG) using a 2D dimensionless parameter space (ζ, τ) (as schematically illustrated in Fig. 2b). ζ is the ratio of the GSFE value at the saddle-point (SP) to the value at the AA configuration, and τ controls flatness of the SP as the second derivative along the line connecting two AA sites (normalized by the GSFE value at the AA configuration). For each point on the (ζ, τ) plane, corresponding to one GSFE candidate, we solve a set of 1D relaxation problems describing the profiles of SDW and DDW at different domain wall orientations (see Supplementary Information S1 for more details on relaxation codes), and extract $\bar{\beta}$. The result is summarized in Supplementary Fig. S3g, and defines a region (green band) in parameter space that would meet the experimental $\bar{\beta} = 1.90$ criterion.

DDW parameter	$E_{S,SDW}$ $/\alpha E_{AA}$	$E_{T,SDW}$ $/\alpha E_{AA}$	$E_{S,DDW}$ $/\alpha E_{AA}$	$E_{T,DDW}$ $/\alpha E_{AA}$	$\bar{\beta}$
$b_{0,0}$	2.64	4.147	5.789	7.519	1.9480
$b_{0,1}$	1.257	1.975	5.628	4.250	0.2944
$b_{0,2}$	-0.127	-0.200	-5.381	-1.049	-0.4872
$b_{0,3}$	0.010	0.0150	2.887	0.252	0.2469
$b_{1,0}$	35.393	55.606	77.790	101.181	-0.2379
$b_{1,1}$	-3.005	-4.720	-0.453	-8.603	0.2722
$b_{1,2}$	0.229	0.359	-2.820	1.104	-0.0856
$b_{2,0}$	-42.950	-67.468	-99.327	-122.838	0.1834
$b_{2,1}$	2.850	4.477	5.834	7.522	-0.1251
$b_{3,0}$	28.682	45.058	66.590	82.285	-0.0791

Table 2: TBG candidates that satisfy $\bar{\beta} = 1.90$.

In the main text we explored the effect of shifting the GSFE parameters toward that region on DDW formations, and observed by 2D relaxation calculations that the experimental observation is reproduced once the TBG GSFE is adjusted as to satisfy $\bar{\beta} = 1.9$ (which is not the case for the existing GSFE parameters in the literature). For completeness, and for practical comparisons with future modelling, we provide the full dependence of the different extracted properties of domain walls on TBG GSFE parameters. Here we assume the mechanical parameters (K and G) remain fixed and follow the literature values mentioned in Table 1 (Supplementary Information S2). Under these conditions we provide in Table 2 expressions for the domain walls parameters (normalized by αE_{AA}, where E_{AA} is the

stacking energy for the AA configuration, and α is the atomic u.c lattice constant). Each parameter is fitted with a third order polynomial in ζ, τ, and we provide $b_{l,m}$ as the coefficient of the $\zeta^l \tau^m$ term.

In Fig. 2d-e we use relaxation calculations (see Supplementary Information S1 for more details) to compare the commonly accepted GSFE version for TBG in literature[31], labeled "Carr et al." with one representative GSFE candidate on the $\bar{\beta} = 1.9$ band (Supplementary Fig. S3g), labeled "moiré constrained GSFE". Here we provide further details about these calculations. Supplementary Figure S4 shows the stacking energy density for the two GSFE versions (literature and moiré constrained GSFE encircled by blue and magenta rectangles respectively). For each GSFE we explore different initial and boundary conditions (IC and BC) leading to the relaxed lattice solutions (second and fourth columns in Supplementary Fig. S4). In the first case (Supplementary Fig. S4a-d), the stacking configuration is forced in a few selected points (dots in a and c), and the IC is spline-interpolated between these points. After relaxation (Supplementary Fig. S4b and d for literature and moiré constrained GSFE respectively) shows the formation of SDWs and DDWs. Interestingly, the moiré constrained GSFE solution shows a symmetric DDW formation reflecting the AB/BA domain wall symmetry. While this is expected, it does not agree with the experimental observation (Fig. 2). Even using an IC which breaks the AB/BA symmetry, by taking the single tuning parameter network as the input into the relaxation calculation (Supplementary Fig. S4e-h) leads to a similar solution (compare Supplementary Fig. S4b-f and d-h). Despite the obscure experimental origin of this symmetry breaking, we can still account for it by adding BC points that break the symmetry,

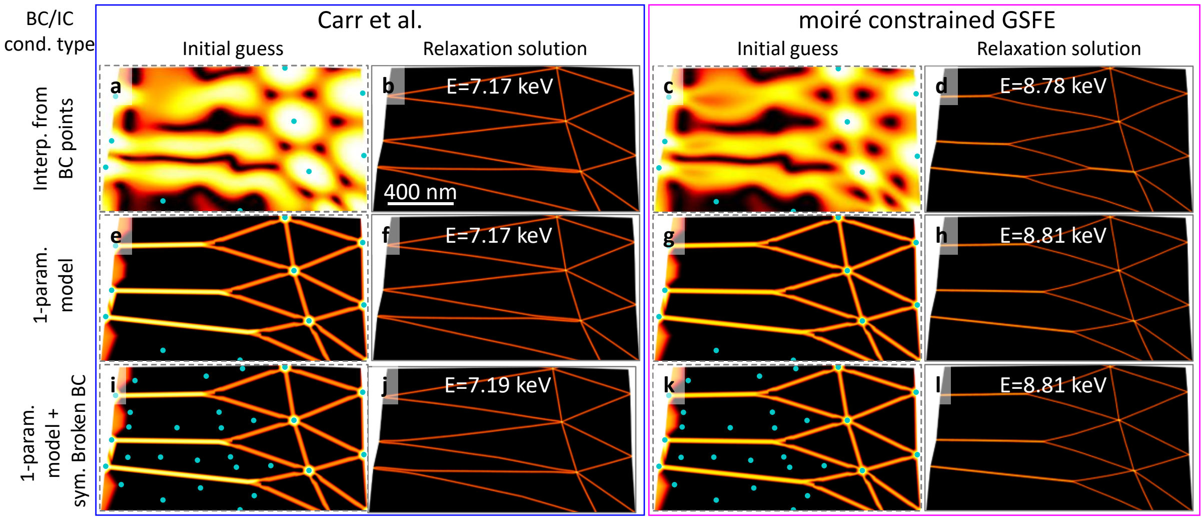

Supplementary Figure S4|Comparing GSFE candidates of TBG through relaxation calculation. a-b, Energy density map of initial guess (**a**) used for the relaxation calculation result (**b**) using the GSFE of Carr et al.[31]. The dots mark points of fixed configuration boundary conditions (BC). The map of **a** was interpolated from the stacking configurations at the dots. **c-d,** Similar to **a-b** but for the moiré constrained GSFE candidate discussed in Fig. 2 and in this section. **e-h,** Similar to **a-d** but with initial conditions set by the single tuning-parameter model with $\bar{\beta}$ (see section text). **i-l,** Similar to **e-h** but with additional BC dots breaking the symmetry between AB and BA domains. All panels share the scale-bar of **b**. Panels **a-b,e-f,i-j** and **c-d,g-h,k-l** share the color-maps of Fig. 2d and Fig. 2e respectively. Total calculated energies after the relaxation process are indicated on each relaxation calculation result, showing the near degenerate nature of the solutions.

as shown in Supplementary Fig. S4i,k leading to the solutions of Fig. 2d-e (Supplementary Fig. S4i and S4l respectively). Note that the solutions have similar total energies pointing to the degenerate nature of the formed domain wall network.

S 5. Defect-induced doping in TDBG stacks

Bilayer graphene flakes that were used to make TDBG stacks for this work were cut in two ways: the 'tear and stack' approach, and by anodic-oxidation lithography (see sample preparation section under Materials and methods). Some of the stacks were measured while still on a transfer slide (stacked on top of a PPC/PDMS stamp/glass slide structure), while others were made into full devices and measured at controlled electrostatic environment. In the histogram of Fig. 3a only data of well-defined electrostatics were used (for the full set of data, with an additional large κ^{-1} valued domains, see Supplementary Information S9 below). In a few of the anodic-oxidation cut stacks, and most pronouncedly in the case presented in Fig. 3e, the stacks seem to have a high level of defect-induced doping. This is apparent from both the enhanced ABAB/ABCA nearfield contrast, as well as by the flattening of the SDW's (as discussed in Fig. 3 and relevant text). The origin of this defect-induced doping is not known, but it is hypothesized to be related to full/partial oxidation of the layer that was cut during the anodic oxidation, perhaps in cases of extremely high humidity levels during the cutting process. Starting from the DFT-D2 approach for TDBG, we examined the evolution of the energy per unit area of the rhombohedral domain, σ, as a function of doping and interlayer bias, based on DFT calculations (as described in Materials and methods). The results are presented in Table 3 below, and they reveal a relatively weak dependence of the curvature on the interlayer bias and n-doping, but a considerable effect of p-doping.

Assuming the change of the curvature in the case of Fig. 3e is mostly from doping, we estimate the doping level from curvature to be roughly $8 \cdot 10^{12}\ cm^{-2}$. At that doping level the GSFE function is defined (as calculated by DFT methods) by the following coefficients: $c_0 = 10.2226$, $c_1 = 6.0451$, $c_2 = -0.4919$, $c_3 = -0.2199$, $c_4 = -0.0037$, $c_5 = -0.0073$ (all in units of $meV/u.c.$). These are the parameters used in Fig. 3b (dashed green) and green dots of Fig. 3f.

$n\ [10^{12}\ cm^{-2}]$	-9.5	-5.7	0	5.7	9.5
$\sigma\ [meV/nm^2]$	3.934	4.445	4.637	1.366	-0.583

$D\ [V/nm]$	0	0.2	0.6	1
$\sigma\ [meV/nm^2]$	4.637	4.499	3.629	2.072

Table 3: σ in as a function of interlayer bias and doping.

S 6. Examples of images for TDBG domains curvature extraction

Supplementary Fig. S5 presents 3 additional representative examples of nearfield (Supplementary Fig. S5a), STM (Supplementary Fig. S5b) and STS (Supplementary Fig. S5c) imaging of TDBG moiré superlattice that were used in constructing the histogram of Fig. 3a. The histogram is based on a total of 31 scans, most of which are not explicitly shown here, but will be provided upon reasonable request.

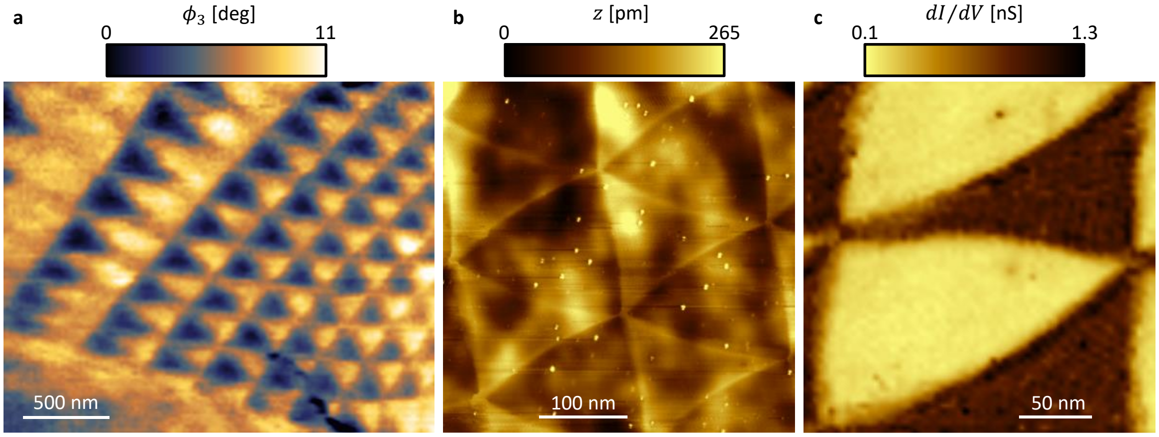

Supplementary Figure S5|Examples of images used for curvature extraction of TDBG domains. a, Mid-IR (940 cm^{-1}) nearfield phase (ABCA – dark, ABAB - bright) similar to Fig. 1c. **b**, STM scan showing ABAB (dark) and ABCA (bright) domains. **c**, STS imaging (ABCA – dark, ABAB – bright) similar to Fig. 1b.

S 7. The 2D soap-bubble model of TDBG

The 2D soap-bubble aims to describe the shape of rhombohedral (ABCA) domains surrounded in a Bernal background (ABAB) as a competition between single domain walls (SDWs), double domain walls (DDWs) and domain area. A preliminary version, restricted to the SDW interface has been presented recently[38]. Here we generalize it to include DDWs, which dominate the structure at the low twist angle limit as well as under external strain. The results of this model were used in the main text (dashed green lines in Fig. 3c-d and solid lines in Fig. 3e), with great agreement with full relaxation calculations except for a narrow angular region in Fig. 3e where DDWs start to form.

We start by describing the interface between ABCA and ABAB in absence of the formation of DDWs. We assume the energy of such an interface is composed of an energy per unit area σ (which is directly extracted from the difference in GSFE values at ABCA and ABAB), and an energy per unit length of a SDW. The latter has an angular dependence of the form $\gamma_1(\varphi) = \left(E^n_{S,SDW}\cos^2\varphi + E^n_{T,SDW}\sin^2\varphi\right)^{\frac{1}{n}}$. First, we assume that the domain wall shape is described by a function $y = f(x)$ with boundary conditions $f(x=0) = f(x=L) = 0$. We can then write an expression for the energy of the domain as: $E = \int_0^L dx\mathcal{E}(x,f,f')$, where the energy functional is $\mathcal{E}(x,f,f') = \sqrt{1+f'^2}\gamma_1\big(\varphi(f')\big) + \sigma f$, and the domain wall orientation angle function $\varphi(f') = \tan^{-1} f'$. After writing Euler-Lagrange equations and substituting $\mathrm{f}' = \tan\varphi$ one can show:

$$x(\varphi) = \tfrac{1}{\sigma}(\gamma_1(\varphi)\sin\varphi + \gamma_1{}'(\varphi)\cos\varphi) + const$$

Furthermore:

$$y'(\varphi) = x'(\varphi)\tan\varphi$$

$$y''(\varphi) = \frac{1}{\cos^2\varphi}x'(\varphi) + \tan\varphi\, x''(\varphi)$$

And one can show that the curvature satisfies:

$$\kappa(\varphi) \equiv \frac{x'y'' - y'x''}{(x'^2 + y'^2)^{\frac{3}{2}}} = \frac{\sigma}{\gamma_1(\varphi) + \gamma_1''(\varphi)}$$

If we approximate $\gamma_1(\varphi)$ to an analytic form of $n = 2$, this would yield an elliptic SDW shape with major (oriented along the shear direction) and minor half axis (A and B respectively) such that:

$$A = \frac{1}{\sigma}E_{T,SDW}, B = \frac{1}{\sigma}E_{S,SDW}$$

For a given GSFE of TDBG within different explored approaches we extract $E_{S,SDW}, E_{T,SDW}$ by solving a 2D relaxation problem such as in Supplementary Fig. S6a, extract A and B and multiply by σ. σ can be evaluated directly from the GSFE as $\sigma = \frac{1}{S_{uc}}(c_4+c_5)$, where $S_{uc}=\frac{\sqrt{3}}{2}\alpha^2$ is the atomic scale unit cell area (introduced due to the unit choice in Table 1 of Supplementary Information S2). Due to the instability of the rhombohedral phase in TDBG, the SDW separating the rhombohedral and Bernal phase is not a stable 1D soliton solution. Instead, it is a 2D structure stabilized by effective pressure, originating from the difference in energy cost of the two phases. As a result, in order to calculate $E_{S,SDW}, E_{T,SDW}$ we have to do 2D relaxation calculations. We define a L_x by L_y rectangular geometry, centered at the origin, and assume a boundary between rhombohedral and Bernal phases. We pin the SDW at two points, $x = 0, y = \pm\frac{1}{2}L_y$, by forcing a saddle point configuration at these points. For any point along the mesh edges far enough from the SDW (50 nm in the results below) we forced a Bernal phase on the right and rhombohedral phase on the left. After relaxation an elliptical arc shaped domain is formed, as shown in Supplementary Figure S6a, suggesting that $\gamma_1(\varphi)$, the angular dependence of the SDW energy cost per unit length mentioned before can indeed be approximated with an exponent of $n = 2$. The major and minor axis of the ellipse (A and B respectively) were extracted by fitting an ellipse (turquoise dashed line in Supplementary Fig. S6a) for DFT-D2, and similarly for all other DFT approaches considered in this work. These geometrical parameters were directly connected to the energy per unit length coefficients as by the above expressions. One should note, though, that this solution is valid as long as DDWs do not start to form, and that once it becomes energetically beneficial to introduce DDWs (due to strain, or do to geometrical limitations) the pure elliptic solution is no longer valid. The angular dependence of the energy cost of a DDW was extracted by 1D calculation as described in Supplementary Information S1, and summarized in Table 1 (Supplementary Information S2), thus extracting all required parameters for the 2D soap-bubble model.

For a given position of the domain vertices (the BAAC stacking locations), the 2D soap-bubble model thus assumes the energy has three contributions: an area term, an energy per unit length term of the SDW and an energy per unit length of the DDW. In the simplest case we treat an equilateral triangular geometry. This will allow us to reach analytic expressions for the solution minimization the energy, and explore the energy functional for the meta-stability discussion as presented in Supplementary Information S9. Under these assumptions the DDW is aligned along the shear direction, and therefore the angular dependence of $\gamma_2(\varphi)$ is reduced to $\gamma_2 = \gamma_2(\varphi = 0)$. We further assume that the SDW segments have an elliptic form with major to minor axis ratio of η. We assume the ellipse is oriented along the shear direction of the SDW, meaning in our simplified geometry, along the lines connecting the vertices, but relax the ellipse dimensions by allowing the minor axis dimension to vary as to minimize the energy. In the case of no DDWs the energy will be minimized by a choice of A, B as dictated by the above Euler-Lagrange equation solution. However, once DDWs start to form that will no longer necessarily be the case. We define two geometrical parameters controlling the domain shape a and χ such that a is the distance from

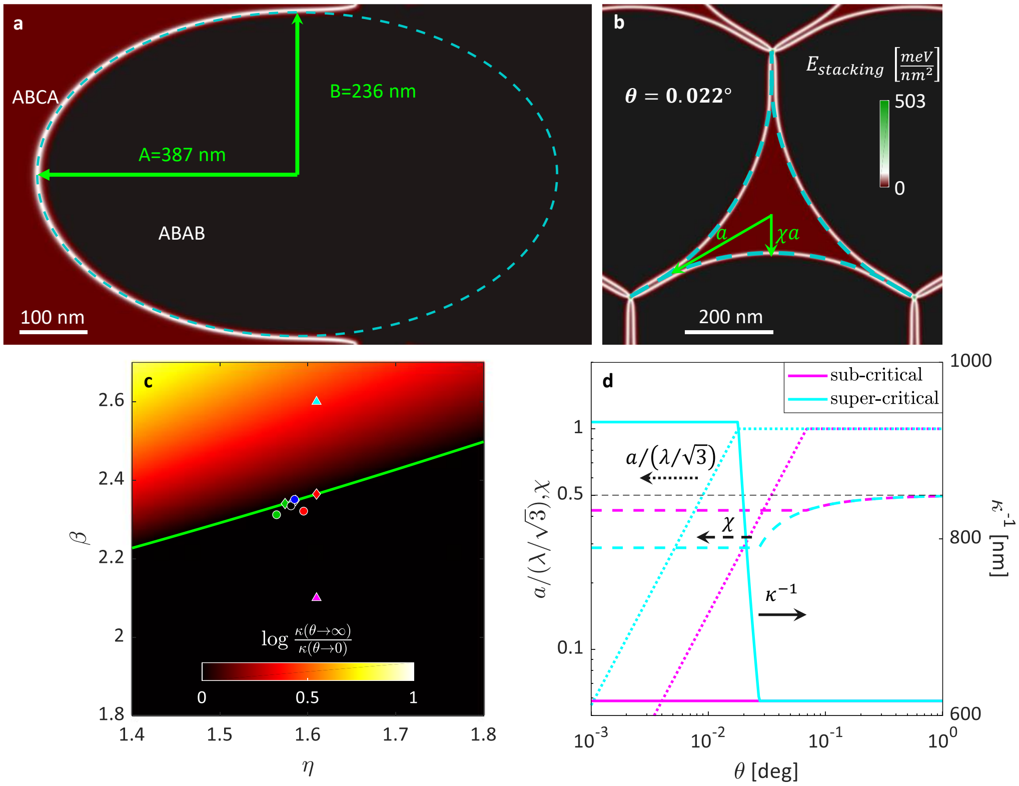

Supplementary Figure S6|Analysis of the 2D soap-bubble model. a, Stacking energy density from a 2D relaxation calculation of the interface between ABCA (left of interface) and ABAB (right of interface) stackings for the GSFE of DFT-D2, yielding an elliptic-arc shaped domain wall. The extracted major (A) and minor (B) axis of the formed ellipse (fit in dashed green line) are directly used as inputs to the 2D soap-bubble model. White double arrow marks shear direction for the SDW dislocation. **b,** Solution of a continuous relaxation model (false color: stacking energy density) and soap-bubble model domain shape (dashed green) for a twist angle of 0.022° for DFT-D2. Geometrical parameters of the soap-bubble model are illustrated. **c,** Phase space revealing a phase transition between a single valued curvature below (the sub-critical regime) a critical line for $\beta_c(\eta) = \sqrt{\eta^2 + 3}$ (green line), and a double asymptote behavior above the critical line (the super-critical regime). The diagram plots the log of SDW curvature asymptotes ratio (at infinite and 0 twist angles) as a function of two dimensionless energy ratios defining the phase-space ($\beta = \gamma_2/E_{S,SDW}$ and $\eta = E_{T,SDW}/E_{S,SDW}$).The markers show the location of the 4 DFT approaches used in this work (with similar color convention as in Fig. 3), plus two artificially added examples (triangles) to demonstrate sub-critical (magenta) and super-critical (cyan) behaviors. Circles are the locations in phase space of the different DFT approaches as extracted directly, and diamonds are fits forced to be along the critical line (see text for more information). **d,** Analysis of the domain geometrical parameters (a, χ) (left y-axis) and resulting inverse curvature (right y-axis) for the sub-critical (magenta) and super-critical (cyan) examples marked in **c**, showing the qualitative difference between the two regimes in the soap-bubble model.

the triangle center to the end point of one of the DDWs and χa is the distance from triangle center to nearest point on the SDW (see notation on representative domain of Supplementary Fig. S6b). λ is the moiré length and the edge of the triangle. Within this notation the shape of one of the 3 domain wall curves defining the domain would be:

$$y(x) = \begin{cases} \dfrac{x}{\sqrt{3}} & 0 \le x \le \dfrac{\lambda}{2} - \dfrac{\sqrt{3}}{2}a \\ y_0 + \sqrt{B(a,\chi,\eta)^2 - \eta^{-2}\left(x - \dfrac{\lambda}{2}\right)^2} & \left|x - \dfrac{\lambda}{2}\right| \le \dfrac{\sqrt{3}}{2}a \\ \dfrac{\lambda - x}{\sqrt{3}} & \dfrac{\lambda}{2} + \dfrac{\sqrt{3}}{2}a \le \lambda \end{cases}$$

Where:

$$B(a,\chi,\eta) = \frac{\frac{3}{4\eta^2} + \frac{1}{4}(1-2\chi)^2}{1-2\chi}a$$

$$y_0(a,\chi,\eta) = \frac{\lambda}{\sqrt{12}} - \chi a - B(a,\chi,\eta)$$

And the parameters are defined within the boundaries:

$$0 < a \le \frac{\lambda}{\sqrt{3}}; \zeta_\eta \le \chi < \frac{1}{2};\ \zeta_\eta = \frac{\sqrt{\eta^2+3}}{2\eta^2}\left(\sqrt{\eta^2+3} - \sqrt{3}\right)$$

Where ζ_η, the bottom limit on χ, is set such that the SDW elliptic segment is tangent to the DDW. Under such assumptions, the total energy is the sum of the SDW and DDW contributions and the domain contributions, $E = E_1(a,\chi,\eta) + E_2(a) + E_\Sigma(a,\chi,\eta)$. Where:

$$E_1(a,\chi,\eta) = 6\eta E_{S,SDW} B(a,\chi,\eta) \int_{\frac{\pi}{2}-\Phi(a,\chi,\eta)}^{\frac{\pi}{2}} d\phi \frac{\sqrt{\eta^4 \sin^2\phi + \left(\frac{E_{T,SDW}}{E_{S,SDW}}\cos\phi\right)^2}}{(1+(\eta^2-1)\sin^2\phi)^{\frac{3}{2}}}$$

$$E_2(a) = 3\gamma_2\left(\frac{\lambda}{\sqrt{3}} - a\right)$$

$$E_\Sigma(a,\chi,\eta) = 3\sigma\left(\sqrt{\frac{3}{4}(1-\eta^{-2})a^2 + B(a,\chi,\eta)^2}\big(\chi a + B(a,\chi,\eta)\big)\sin\Phi(a,\chi,\eta) - \eta B(a,\chi,\eta)^2\left(\frac{\pi}{2} - \tan^{-1}(\eta\cot\Phi(a,\chi,\eta))\right)\right)$$

$$\sin\Phi(a,\chi,\eta) = \sqrt{\frac{\frac{3}{4}a^2}{\frac{3}{4}(1-\eta^{-2})a^2 + B(a,\chi,\eta)^2}}$$

If we further assume $\eta = \frac{E_{T,SDW}}{E_{S,SDW}}$, as would be the case for the energy minimizing shape of a SDW in the absence of formation of DDWs, then the expression for $E_1(a,\chi,\eta)$ can be simplified to the analytic form:

$$E_1(a,\chi,\eta) = 6\eta E_{S,SDW} B(a,\chi,\eta)\left(\frac{\pi}{2} - \tan^{-1}(\eta\cot\Phi(a,\chi,\eta))\right)$$

This defines a two dimensional phase-space for the domain energy, as presented in the Supplementary Fig. S6c and specific examples of sub-critical and super-critical twist angle dependence presented in Supplementary Fig. S6d. Mapping the phase-space, one can show that there is a second order phase transition as a function of $\beta \equiv \frac{\gamma_2}{E_{S,SDW}}$, across $\beta_c(\eta) = \sqrt{\eta^2+3}$. Below this line, the sub-critical regime (represented here by the magenta triangle in Supplementary Fig. S6c), there is a single valued curvature of the SDW (as a function of twist angle) satisfying $\kappa_\infty^{-1} = \eta^2 \frac{E_{S,SDW}}{\sigma}$. Above the line, the super-critical regime (represented here by the cyan triangle in Supplementary Fig. S6c), the curvature covers a span of values between two asymptotic values. For a large twist angle the system converges to a similar domain structure as in the sub-critical regime, but at the low twist angle limit, a different solution emerges satisfying the ratio: $\frac{\kappa_{\theta\to 0}^{-1}}{\kappa_{\theta\to\infty}^{-1}} = 1 + \frac{1}{\beta_c}\frac{\beta-\beta_c}{1-\frac{\sqrt{3}}{\eta}\left(\frac{\pi}{2}-\tan^{-1}\frac{\sqrt{3}}{\eta}\right)}$. The two regime also differ in terms of the path the energy minimizing point takes on the (a,χ) plane as a function of twist angle, as shown in Supplementary Fig. S6d. In the super-critical regime (cyan case in Supplementary Fig. S6d), the energy reaches its minimal value on the edges of the phase-space. The super-critical of Supplementary Fig. S6d reveal three regimes as a function of twist angle. The first regime, of SDW deformation, is revealed for large twist angles, while $\lambda < \lambda_{c2} = \frac{2}{\beta_c}\kappa_{\theta\to\infty}^{-1}$. In that regime the energy minimizing point travels along the right edge of the (a,χ) plane at $(a,\chi) = \left(\frac{\lambda}{\sqrt{3}}, \frac{1}{2} + \frac{\sqrt{3}}{2\eta}\left[\sqrt{\left(\frac{\beta_c\lambda_{c2}}{\eta\lambda}\right)^2 - 1} - \frac{\beta_c\lambda_{c2}}{\eta\lambda}\right]\right)$. As λ approaches λ_{c2} , χ approaches ζ_η, the bottom edge of the (a,χ) plane. For intermediate values of $\lambda_{c2} \le \lambda \le \lambda_{c1} = \frac{2}{\beta_c}\kappa_{\theta\to 0}^{-1}$ the energy will be minimized for a fixed point of $(a,\chi) = \left(\frac{\lambda}{\sqrt{3}}, \zeta_\eta\right)$. In this narrow angular regime, the 'self-similar regime', the domain expands self-similarly as the twist angle decreases. As the twist angle further decreases, (a,χ) do not change with λ, staying fixed at $(a,\chi) = \left(\frac{\lambda}{\sqrt{3}}, \zeta_\eta\right)$. In this regime, of DDW formation, the rhombohedral domain no longer changes and remains fixed while DDWs extend from its vertices, effectively traveling to the left along the bottom edge of the (a,χ) plane.

In contrast, in the sub-critical regime (magenta line in Supplementary Fig. S6d), when $\beta \le \beta_c$, the SDW deformation stage ends before reaching the bottom edge of the (a,χ) plane. Throughout this entire regime χ satisfies: $\chi(a) = \frac{1}{2} + \sqrt{\left(\frac{\kappa_{\theta\to\infty}^{-1}}{\eta^2 a}\right)^2 - \frac{3}{4\eta^2}} - \frac{\kappa_{\theta\to\infty}^{-1}}{\eta^2 a}$. Once λ reaches $\sqrt{3}a_c$ where $a_c = \frac{2}{\beta_c^2}\left(\frac{\beta}{\sqrt{3}} - \frac{\sqrt{\beta_c^2-\beta^2}}{\eta}\right)\kappa_{\theta\to\infty}^{-1}$, DDW start to form, the energy will be minimized for a fixed point of $(a,\chi) = (a_c, \chi(a_c))$ and the rhombohedral domain will no longer change with decreasing twist angle.

It is important to note that the soap-bubble model agrees very well with the full relaxation solution, as shown in the main-text and Supplementary Videos 2-5. However, for the super-critical case, there is a narrow angular range between the 'SDW deformation' regime to the 'DDW formation' regime, in which the two models disagree. The full relaxation solution does not show a clear self-similar expansion, as predicted by the soap-bubble model in the super-critical regime. This is probably due to the simplistic

representation of the DDW in the soap-bubble model, that doesn't consider a realistic gradual formation of a DDW by collapsing two single SDWs.

Surprisingly, all 4 DFT approaches considered in this work for describing TDBG fall extremely close to the critical line (see circle in Supplementary Fig. S6c relative to green line), with β within about 1% of β_c. Therefore, and due to higher numerical precision of the calculation of $E_{s,DDW}$ (from 1D models) and $E_{s,SDW}$ (from moiré domain relaxation calculations as in Fig. 3c-d) compared to extracting the elliptic arc shape of a SDW (as in Supplementary Fig. S6a), in our modelling of TDBG in this work we assumed all 4-models (with parameters listed in Table 1 of Supplementary Information S2) fall on the critical line (diamonds in Supplementary Fig. S6c) but forcing the extracted $E_{s,DDW}$ and $E_{s,SDW}$.

S 8. Moiré domains deformation under strain

As shown experimentally, moiré patterns in the low twist angle limit are highly susceptible to strain, resulting in some cases in elongated 1D structures and formation of double domain walls. In this section we will present an analytical description of the origin of this behavior.

We restrict our discussion to the case of a fixed external strain, which in its most general form is assumed to be a 2D symmetric tensor: $\overleftrightarrow{\epsilon} = \begin{pmatrix} \epsilon_{xx} & \epsilon_{xy} \\ \epsilon_{xy} & \epsilon_{yy} \end{pmatrix}$. The strain can be written in the following way:

$$\overleftrightarrow{\epsilon} = \epsilon_c I + \epsilon_s(\cos\phi_s\,\sigma_x + \sin\phi_s\,\sigma_z)$$

Where $\epsilon_c = \frac{\epsilon_{xx}+\epsilon_{yy}}{2}$ is a pure dilation strain and $\epsilon_s = \sqrt{\left(\frac{\epsilon_{xx}-\epsilon_{yy}}{2}\right)^2 + \epsilon_{xy}^2}$ is a shear strain term. In this notation ϕ_s encodes information about the direction of the shear strain. σ_x and σ_z are Pauli's matrices in the standard notation. Before any relaxation takes place the displacement field will take the form (setting without loss of generality the displacement field at the origin to 0):

$$\boldsymbol{u}(\boldsymbol{r}) = \epsilon_c \boldsymbol{r} + \epsilon_s(\cos\phi_s\,\sigma_x + \sin\phi_s\,\sigma_z)\boldsymbol{r}$$

When ϵ_s is sufficiently large a critical behavior emerges, in which for a finite $\epsilon_s = s_c$ the moiré unit cell collapses to form a 1D structure, as indeed observed experimentally. This behavior can be seen from deriving expressions for the moiré unit cell lattice vectors, V_1, V_2, which take the form (following the notation of the previous section):

$$V_j = \frac{\alpha}{\Delta}\Big((1-(1-\mu)\epsilon_c)R_{\psi_j+\theta} - (1+\delta)(1+\mu\epsilon_c)R_{\psi_j} + \epsilon_s(1-\mu)R_{\phi_s-\psi_j-\theta} + \epsilon_s\mu(1+\delta)R_{\phi_s-\psi_j}\Big)\begin{pmatrix}1\\0\end{pmatrix};\, j = 1,2$$

Where $\psi_1 = \theta_0$, $\psi_2 = \theta_0 + \frac{\pi}{3}$, $R_\psi = \begin{pmatrix}\cos\psi & -\sin\psi \\ \sin\psi & \cos\psi\end{pmatrix}$ is the rotation matrix by an angle ψ, and:

$$\Delta = (\epsilon_c(1-\mu)-1)^2 - (1-\mu)^2{\epsilon_s}^2 + (1+\delta)^2((1+\epsilon_c\mu)^2 - {\epsilon_s}^2\mu^2) - 2(1+\delta)\big(1+(2\mu-1)\epsilon_c - (1-\mu)\mu({\epsilon_c}^2 - {\epsilon_s}^2)\big)\cos\theta$$

We further define the two emerging periods of the moiré super-lattice, $\lambda_j \equiv |V_j|$. From this expression, it is clear that as $\Delta \to 0$, the moiré unit cell would diverge. We define the quantity s_c, such that $\epsilon_s = s_c$ leads to $\Delta = 0$. s_c should be regarded as a critical shear strain. Let's examine the area of the moiré unit cell as

a figure of merit of this critical behavior. After some further derivation, one can show that it takes the form:

$$S_{moiré} \equiv |V_1 \times V_2| = \frac{\sqrt{3}}{2}\alpha^2 \frac{(s_c^2 - \epsilon_s{}^2)^{-1}}{(1-\mu)^2 + (1+\delta)^2\mu^2 + 2(1+\delta)(1-\mu)\mu\cos\theta}$$

Supplementary Figure S7 explores the structural dependence of the moiré super-lattice. Supplementary Fig. S7a-c provide a specific example of the collapse of a unit cell as ϵ_s approaches s_c for pure shear of a mismatched system (using $MoSe_2/WSe_2$ parameters as a representative of such a system). Supplementary Fig. S7a explores the moiré cell collapse for a strain oriented along the x-axis, Supplementary Fig. S7b presents the unit cell area as a function of s_{shear} and strain angle (however, curves of all angles collapse). Note that while neither $S_{moiré}$, Δ nor s_c depend on strain direction (from the above expressions), the exact nature of the collapse as well as relaxation calculations (Supplementary Fig. S7d-f) are affected by these details.

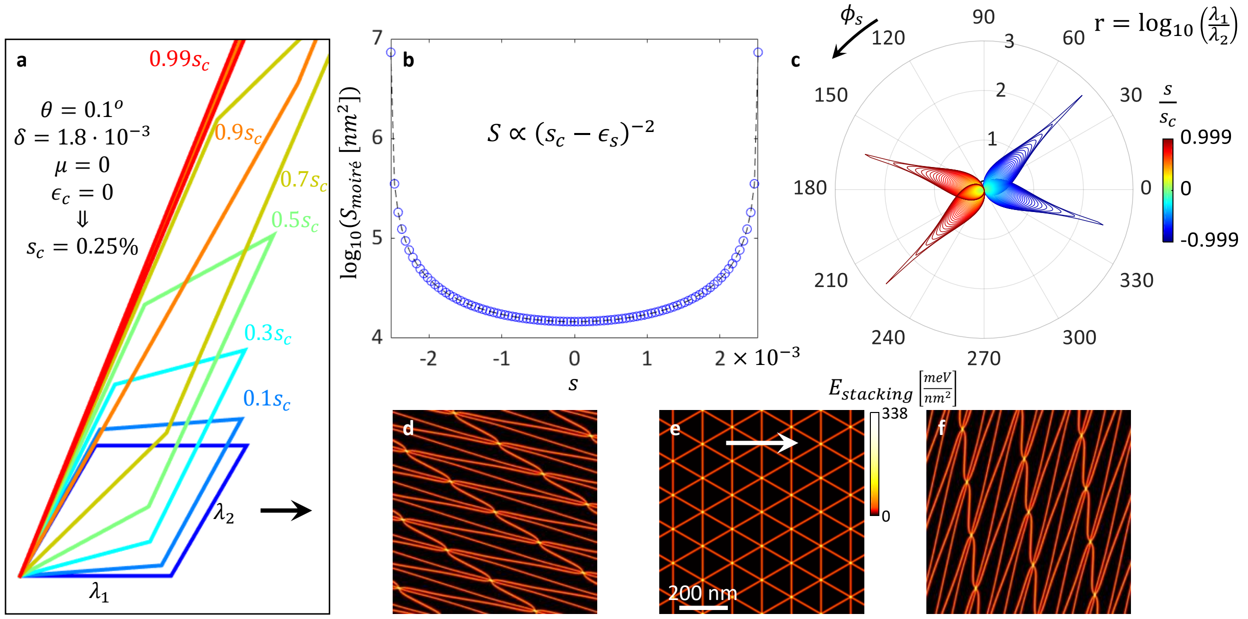

Supplementary Figure S7 | Critical structural dependence of the moiré super-lattice on pure shear strain. **a-c,** Moiré unit cell collapse due to shear strain for mismatched system with $\theta = 0.1^o, \delta = 1.8 \cdot 10^{-3}$, $\mu = 0$, $\epsilon_c = 0$ (see text for details), showing the collapse of the unit cell to a 1D structure toward a critical shear strain value of 0.25% (**a** – strain direction of example marked by arrow), yielding a diverging unit cell area independent of strain orientation (**b**). **c,** Polar plot of ratio of two unit-cell lattice spacings (on a log scale). Color indicates the shear strain value relative to the critical strain. The strong directionality of the pattern suggests that the resulting collapsed patterns depend on strain orientation. **d-f,** Stacking energy density from 2D relaxation calculations of uniaxially strained 0.1° TBG (assuming a Poisson ratio of 0.22), for 3 different strain values: -0.14% (**d**), 0% (**e**), 0.14% (**f**). White arrow in **e**: The strain orientation for **d-f**.

In order to get some intuition about the expression let's simplify matters by examining a system with no mismatch (therefore $\delta = 0$), such as a homo-bilayer structure. If we further assume for simplicity that one layer is rigid, i.e. $\mu = 0$, we get:

$$s_c^2 = \epsilon_c{}^2 + 4(1-\epsilon_c)\sin^2\frac{\theta}{2};\ S_{moiré} = \frac{\sqrt{3}}{2}\alpha^2(s_c^2 - \epsilon_s{}^2)^{-1}$$

From this expression it is clear that the critical behavior will be observed only if ϵ_s approaches $\sqrt{\epsilon_c{}^2+4(1-\epsilon_c)\sin^2\frac{\theta}{2}}$, and therefore the dilation strain has a stabilizing effect on the unit-cell, preventing its collapse.

So far this discussion was somewhat hypothetical, since ϵ_c and s were treated as independent. In practice, we need to do a more careful analysis. First, the strain mentioned above is the relative inter-layer strain, composed of the strain tensor of each layer composing the bilayer structure. Second, the strain tensor of layer i has the form:

$$\overleftrightarrow{\epsilon_i} = \frac{1-\nu_i}{2} S_i \begin{pmatrix} 1 & 0 \\ 0 & 1 \end{pmatrix} + \frac{1+\nu_i}{2} S_i(\cos\phi_s\,\sigma_x + \sin\phi_s\,\sigma_z)$$

Where ν_i is the Poisson ratio of that layer. If we assume the two layers are strained along the same direction (both have the same ϕ_s) we would get a relative strain tensor of a similar form:

$$\overleftrightarrow{\epsilon} = \frac{1-\nu}{2} S \begin{pmatrix} 1 & 0 \\ 0 & 1 \end{pmatrix} + \frac{1+\nu}{2} S(\cos\phi_s\,\sigma_x + \sin\phi_s\,\sigma_z)$$

For $S = S_2 - S_1, \nu = \frac{\nu_2 S_2 - \nu_1 S_1}{S_2 - S_1}$, and we can continue with the same analysis, as before with $\epsilon_s = -\nu S$, $\epsilon_c = S$. For the simplest case of a homo-bilayer structure in the low twist angle limit, such that $\delta = 0, \theta \ll \nu = \nu_1 = \nu_2$ we get a critical strain of approximately $\frac{2}{\sqrt{\nu}}\sin\frac{\theta}{2}$. This realistic critical behavior in twisted bilayer graphene is demonstrated in Supplementary Fig. S7d-f and in Supplementary Video 6.

S 9. Large rhombohedral domains as meta-stable states

Figure 3a presented a histogram of radii of curvature of TDBG rhombohedral domains for cases of controlled electrostatic environment near CNP. Throughout the fabrication process, before the stacks were contacted with metallic electrodes, and specifically for stacks that were cut with anodic oxidation (see methods section), we persistently observed isolated large rhombohedral domains with significantly larger κ^{-1} values, up to $\kappa^{-1} = 5 \cdot 10^3\ nm$. Supplementary Fig. S8a extends the histogram of Fig. 3a to include these cases as well (red bins). These latter large domains are stable at room temperature but disappear under thermal annealing, as shown in Supplementary Fig. S8b-c, suggesting they are meta-stable states. In this example we observe the annihilation of large rhombohedral domains by vacuum thermal annealing (at 350°C) which is a common step in the fabrication process of vdW heterostructures. Supplementary Fig. S8b presents the nearfield phase of a TDBG device before thermal annealing, showing a few large ABCA domains (dark regions marked with red arrows) and a strained structure. After annealing (Supplementary Fig. S8c) the large domains vanish, as the overall moiré pattern becomes more uniform, and seems to indicate a reduced level of strain (except for the left part, where a mechanical rotator was placed, clearly deforming the pattern, as expected). This behavior, of annihilation of large rhombohedral domains by thermal annealing, seems to be a common theme in these structures. A more vivid demonstration of the meta-stability of these large domains is presented in the insets Supplementary Fig. S8d-f, which are three sequential mid-IR nearfield scans of the same region (see methods). Due to optical field enhancement by the metallic tip the region under the tip experiences excessive heating, which likely drives this transition. The light-induced collapse of one of the domains (red arrows in inset Supplementary Fig. S8e) suggests the scanning tip acted as a local thermal annealer attesting to the meta-stable nature

of the domain. This can be seen by comparing domain structure in Supplementary Fig. S8e above arrows (before collapse) to an earlier scan (Supplementary Fig. S8d) and below arrows (after collapse) to a later scan (Supplementary Fig. S8f).

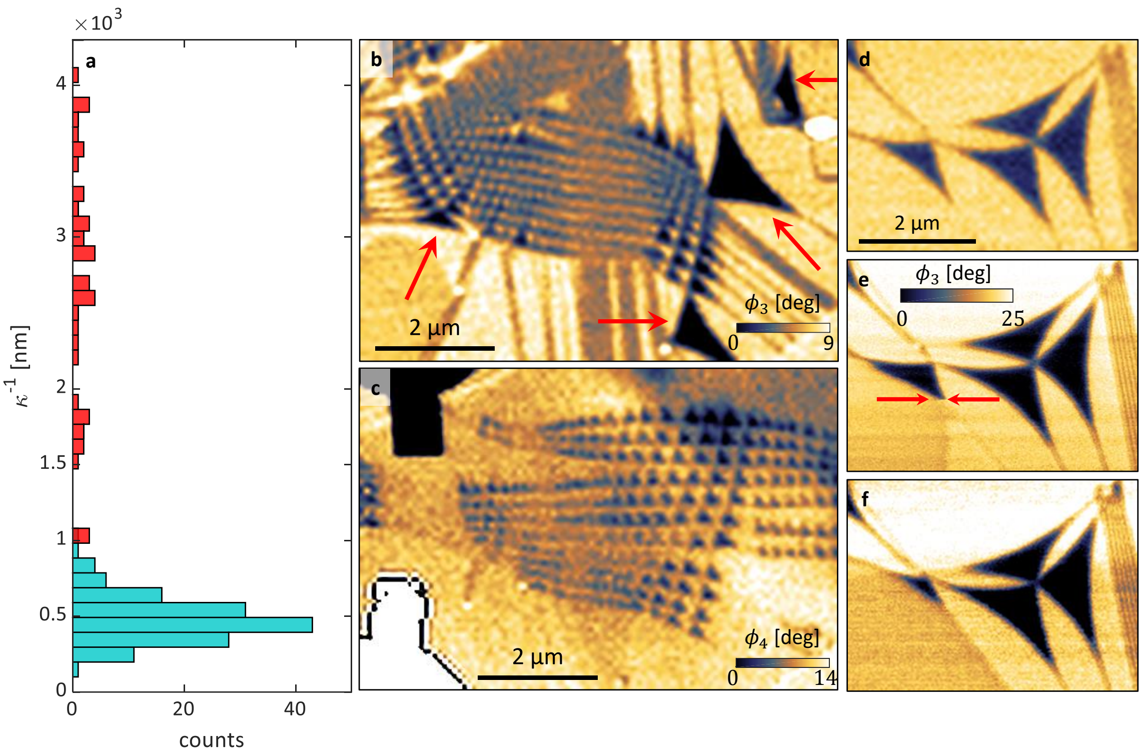

Supplementary Figure S8|Experimental indication of meta-stability of rhombohedral domains in TDBG. a, Curvature histogram across all domains measured in this work, including those away from CNP. On top of the main cluster discussed in main-text (turquoise bins) there is a tail of large domains (red bins), that appear to be meta-stable. **b-c,** Effect of vacuum thermal annealing of a device shows a dramatic effect on rhombohedral domains pointing to their meta-stability. Before annealing (**b**) there exist large rhombohedral domains (dark) which completely vanish after annealing (**c**). The annealing also leads to strain relief which makes a more regular moiré super-lattice closer to the ground state. The added structure on the left top and bottom of (**c**) are rotators placed on top of the stack for unrelated purposes. **d-f,** 3 subsequent nearfield phase imaging scans of meta-stable ABCA domains, representing the large κ^{-1} tail of the histogram, and demonstrating meta-stability by observing tip-induced annealing of one of the domains mid-scan**.** Red arrows (**e**) mark a sudden change in domain shape as the tip scans from top to bottom, which in later (**f**) reveals a collapsed domain. **d-f** share a color-map and scale-bar.

Attempting to pin-down the source of this meta-stability, one can come up with a few possible explanations. One obvious explanation could be that the stacks are charged, and therefore the ground-state curvature deviates from the CNP prediction presented in Fig. 3. If, for instance, during the anodic oxidation process the top graphene layer was oxidized, that would result in a charge transfer that would lead to a defect-induced doping, as we believe is the case for Fig. 3e. However, the meta-stability of the large domains, as indicated by their collapse under heating, requires a different mechanism.

Let's further explore meta-stability in this system computationally by a closer look at the relaxation process through the calculation. We track the domain walls of the system through different gradient descent steps of the simulation as it approaches toward an energy-minimizing solution. This mimics the actual relaxation process, as domain formation is driven by local forces governed by elastic and stacking energy terms. Supplementary Fig. S9a plots the evolution of the rhombohedral domain through the last phases of the 2D relaxation calculation of Fig. 3d. The domain wall is tracked for a given step and assigned a color, corresponding to the instantaneous total energy at that iteration step. Initially a straight domain wall is formed (not shown for brevity). Only near convergence does the domain wall bend inward (decreasing κ^{-1}) to further decrease the energy. At some point two SDWs partially collapse to form a DDW, before converging to the ground-state. Observing how sensitive κ^{-1} is to the energy density, one can expect that a small energy barrier could readily trap the system in a meta-stable state with, in principle, an arbitrarily large κ^{-1} value. We can imagine the path in phase-space the system takes

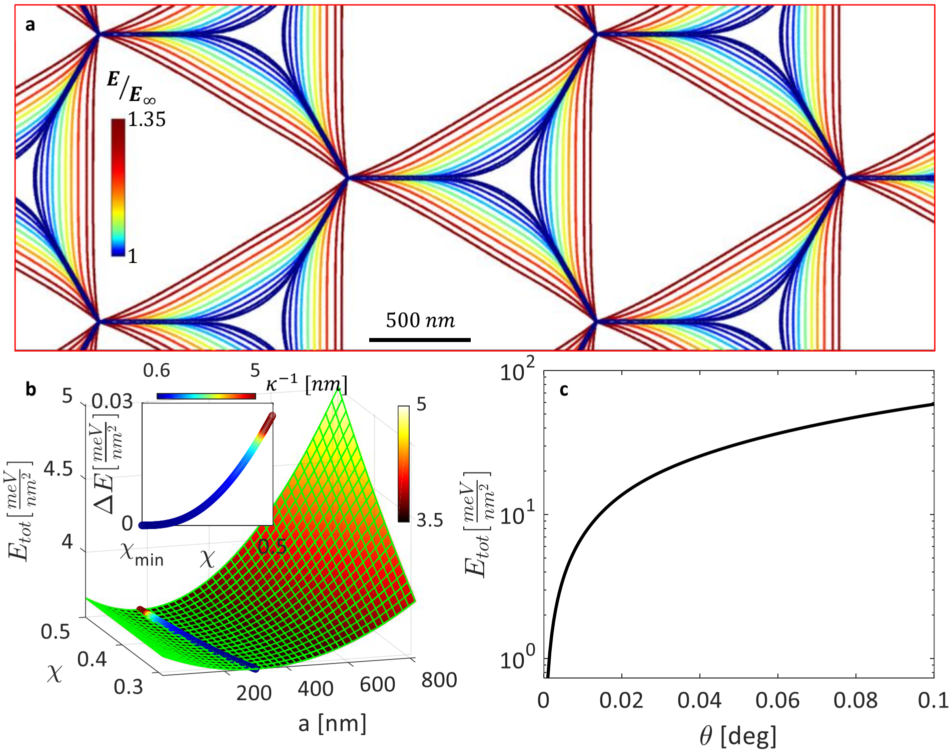

Supplementary Figure S9|Modelling meta-stability of rhombohedral domains in TDBG. a, Tracking domain wall shapes through the final energy minimization iterations of the calculation of Fig. 1d**.** A sharp drop in κ^{-1} is observed as the system relaxes to its ground state. Each configuration is colored according to its total energy normalized to the ground-state energy (indicated by color-map). **b,** Total energy landscape within the 2D soap-bubble model detailed in Supplementary Information S9 for a twist angle of 0.01°, revealing a steep direction (along a) and a shallow direction (along χ). Colored circles (Inset and on landscape) mark follow the shallow valley and indicate the domain radius of curvature at a given point. The parameters of DFT-D2 were used in this example (see Supplementary Information S2). **c,** Averaged (over moiré unit-cell) energy density of TDBG as a function of twist angle within the 2D soap-bubble model.

through the relaxation process. Supplementary Fig. S9a suggests that close to the ground state the energy landscape is shallow, showing minimal changes in energy, while the curvature changes dramatically. We can further visualize this landscape by the presented soap-bubble model, as shown in Supplementary Fig. S9b. Such an approach suggests, as expected, the existence of a steep direction (mostly along parameter a) and a shallow direction (mostly along parameter χ) in the energy landscape. The radius of curvature strongly changes (indicated by colored circles) along the shallow valley from a straight domain walls on one end of the valley (for maximal χ) down to small κ^{-1} value of the true ground state at the other end of the valley, all throughout an energy drop of 30 $\mu eV/nm^2$. One can expect that if there were an energy barrier of that scale, it could readily result in a meta-stable state with large κ^{-1} value. One possible source of such an energy barrier would be twist-angle inhomogeneity or strain. Supplementary Fig. S9c compares the total energy averaged over a moiré unit-cell as a function of twist angle within the 2D soap-bubble model (and the parameters of the DFT-D2 approach listed before). One finds that for an experimentally observed twist angle variation the resulting energy landscape is expected to be sufficiently disordered to compete with relaxation processes along the shallow valley, and account for the required energy barrier to trap the system in a meta-stable state with large κ^{-1} values. In that sense, thermal annealing has two effects: It may provide the activation energy to push the system beyond a barrier, or alternatively, it relieves strain and leaves the system more uniform, thus removing energy barrier altogether.
